# Oral Contraceptives and Renal Water Handling: A diurnal study in young women

**DOI:** 10.14814/phy2.13547

**Published:** 2017-12-12

**Authors:** Charlotte Graugaard‐Jensen, Gitte M. Hvistendahl, Jørgen Frøkiær, Peter Bie, Jens Christian Djurhuus

**Affiliations:** ^1^ Department of Urology University Hospital of Aarhus Aarhus N Denmark; ^2^ Department of Clinical Physiology and Nuclear Medicine University Hospital of Aarhus Aarhus N Denmark; ^3^ Institute of Molecular Medicine University of Southern Denmark Odense Denmark; ^4^ Institute of Clinical Medicine University Hospital of Aarhus Aarhus N Denmark

**Keywords:** Arginine vasopressin, diuresis, diurnal rhythm, oral contraceptives, oxytocin, prostaglandin E2

## Abstract

To test the hypothesis that use of oral contraceptives (OC) changes diurnal variation in fluid balance mechanisms including blood pressure, secretion of vasopressin and oxytocin, and renal water and electrolyte excretion. Fifteen naturally cycling (NC) women in mid‐follicular phase and 11 long‐term OC users were included in a 24‐h standardized inpatient study for measurements of vasopressin, oxytocin, sodium, and osmolality in plasma as well as urinary excretion of electrolytes, aquaporin‐2, and prostaglandin E2. Blood pressure and heart rate were monitored noninvasively. Plasma vasopressin showed circadian rhythm (*P* = 0.02) and were similar in both groups (*P* = 0.18) including nighttime increases (*P* < 0.001). There was no circadian rhythm in plasma oxytocin within (*P* = 0.84) or between groups (*P* = 0.22). OC users had significantly lower plasma osmolality (Δosm: 3.05 ± 0.29 mosm/kg, *P* = 0.04) and lower plasma sodium (ΔNa^+:^ 0.91 ± 0.09 mmol/l, *P* = 0.05). The two groups showed similar nighttime decreases in diuresis (1.08 ± 0.04 mL/(kg·h), *P* < 0.001) and increases in urine osmolality (109 ± 9 mosm/kg, *P* = 0.02), but similar rates of excretion of Aquaporin‐2, prostaglandin E2 and sodium. Nighttime decreases in mean arterial pressure of approximately 13% were significant in both groups (*P* < 0.001), but 24‐h average mean arterial pressure was significantly higher in OC users than in controls (+4.7 ± 0.4 mmHg, *P* = 0.02). Packed cell volumes were similar between groups (*P* = 0.54). OC does not change the diurnal patterns of renal fluid excretion, but resets the osmoreceptors for vasopressin release and leads to a significant increase in arterial blood pressure.

## Introduction

The influence of oral contraceptives on blood pressure and fluid balance is not fully elucidated and several homeostatic mechanisms are potentially involved.

Studies in young women have found that estrogens reset the osmoreceptors for thirst and AVP release during high estrogen phases (the luteal phase/oral contraceptives (OC)) (Stachenfeld et al. 1999; Calzone et al. 2001). Yet another study has shown that the well‐established nighttime increase in vasopressin (AVP) was attenuated in premenopausal women taking OC (Kostoglou‐Athanassiou et al. 1998). In postmenopausal women, steroid ovarian hormones influence secretion of both oxytocin and AVP. Estrogen was found to increase AVP concentration, whereas progesterone suppressed the vasopressin concentration (Bossmar et al. 1995). Previously, we have been focusing on the impact of estrogen on the renal regulation of urine production. Eight women were investigated both in the follicular and the luteal phase of the menstrual cycle. There was a preserved nocturnal increase in AVP with both low and high levels of estrogen and no difference in the concentration of AVP, diuresis, urine osmolality, and excretion of aquaporin 2 (AQP2) (Graugaard‐Jensen et al. 2008). However, this could be due to possibility that the minor difference in estrogen between the two phases of the normal menstrual cycle was too subtle to reveal an effect.

Exogenous oxytocin is clearly antidiuretic and can cause water intoxication if administered to pregnant women during delivery (Rasmussen et al. 2004; Wang et al. 2000). The affinity of oxytocin to the V2 receptor is, however, much lower than that of vasopressin (Joo et al. 2004). Whether oxytocin possesses an antidiuretic effect under normal physiological conditions is not fully elucidated. Only a few studies have examined the diurnal rhythm of oxytocin, and the results are inconclusive, concerning the influence on urine production (Kostoglou‐Athanassiou et al. 1998; Forsling et al. 1998; Landgraf et al. 1982). High estrogen levels, as present in OC users, have been found to increase the concentration of oxytocin (Kostoglou‐Athanassiou et al. 1998; Amico et al. 1981; Mitchell et al. 1981) but whether this is sufficient to induce antidiuresis is still unknown.

The role of urinary prostaglandin E2 (PGE2) in renal water handling is still unclear. The urinary excretion of PGE2 is thought to reflect renal prostaglandin production with an effect on both sodium channels (Ando and Asano 1995) renal hemodynamics, and AVP‐regulated water reabsorption (Anderson et al. 1975; Berl et al. 1977). Excess nocturnal PGE2 excretion is seen in patients with nocturnal polyuria provoked by sleep‐deprivation (Mahler et al. 2012; Haack et al. 2009) and in desmopressin‐resistant nocturnal polyuria in children (Kamperis et al. 2012). In rats, estrogen affects the biosynthesis of PGE2 in the kidney (Chang 1989). Whether estrogen affects the renal PGE2 production in women is uncertain. Several studies have found that urinary PGE2 excretion was stable during the normal menstrual cycle (Stachenfeld et al. 2003; Stratton et al. 1986), whereas others have shown that the urinary excretion of PGE‐2 is elevated by ovarian stimulation (Moutquin et al. 1984). The latter could indicate that PGE‐2 may modulate the diurnal urine regulation in women taking OC's.

This study was designed to investigate, whether the enhanced estrogen concentrations obtained with OC use (third generation) affect the diurnal regulation of urine production with focus on the relationship between AVP, oxytocin, AQP2, and PGE2. Women in midfollicular phase were compared to women taking oral contraceptives. We hypothesized that oral contraceptives decrease the concentration of AVP, attenuate the nocturnal increase in AVP, and increase the concentration of oxytocin, which subsequently will modulate the outcome of urine production. In addition, we assessed whether it was possible to compare the in‐patient study design to everyday life using the volunteer's home recordings.

## Materials and Methods

The regional Committee on Biomedical Research Ethics approved this study protocol, and informed consent was obtained from all participants.

### Study subjects

Fifteen healthy nonsmoking natural cycling women (NC) and 11 healthy nonsmoking long‐term OC (3. generation with 20 *μ*g estradiol and gestoden/desogestrel) users were recruited by advertisement at the University of Aarhus. Part of the data obtained from the 15 young women has previously been published (Graugaard‐Jensen et al. 2008; Graugaard‐Jensen C et al. 2014). Inclusion criteria were age 20–35 years, BMI < 30, a normal physical examination including blood pressure measurements and for the women without OC use, a regular menstrual cycle (26–33 days). Participants had normal plasma‐creatinine, sodium, potassium, hemoglobin, and a normal urine analysis. A complete bladder emptying upon voiding was determined by postvoid ultrasound estimation. There were no reports of nocturia or any other urological complaints, no previous surgery, no history of severe illnesses, or use of any medication except for the oral contraceptives.

### Study design

The experimental procedure consisted of a 24‐h in‐patient study under standardized conditions regarding sodium and water intake. The natural cycling women were investigated in midfollicular phase (7–10 days after their last period began), and the women who used OC were investigated during the pill‐period. A 3‐day frequency/volume chart (FVC) was obtained from all participants. Time and volume (mL) of each micturition and of each fluid intake were recorded. Intake of fluid and food were ad libitum*,* and the subjects were asked not to alter their habits during the home recordings. The time of rising and the bedtime were noted.

Two days prior to the admission, the participants were fluid preconditioned to 30 mL/kg body weight 24/h. The participants were told to refrain from alcohol and caffeinated beverages and heavy physical activities 24 h before the admission. The participants were admitted at 7 am. An intravenous access was established in a cubital vein of the nondominant arm. During the experimental period, diet and fluid intake were standardized as directed by a clinical dietitian (sodium 3 mmol/kg body weight 24/h and 30 mL tap water kg^−1^ body weight 24/h distributed equally during the day, 2/5 before 12:00, 2/5 from 12:00 to 18:00 and 1/5 from 18:00 until bedtime). Meals were served at 8:00, 12:00, and 18:00 h. Activity was allowed from arrival until 23:00. Sleep during daytime was not allowed. The OC was taken just before bedtime. From 23:00 the participants had to be in supine position. Physical activity, food, and fluid intake during the night were not allowed.

Blood samples were drawn every 6 h until 20:00, thereafter every 3 h. The Participants were seated in a chair 30 min before blood sampling during the day (8:00–23:00 h). During the night (23:00–08:00 h) samples were taken in the supine position. Care was taken not to wake up the participants during blood sampling at night. The blood samples were replaced by isotonic saline. Blood samples were transported on ice, centrifuged at +4°C. They were stored at −80°C unless immediately analyzed. Blood samples were analyzed for AVP, creatinine, osmolality, and sodium. Estradiol, progesterone, and testosterone were measured only once (day 1, 08:00 h).

Urine was collected with 3‐h intervals following spontaneous voiding. Participants were asked to empty their bladder just after blood sampling during the day, before bedtime and upon wakening in the morning. All other voiding was free at will. Nocturnal urine was collected in a bottle during the night and the participants were asked to empty their bladder in the same bottle at 08:00, day 2. Urine volume and the concentration of creatinine, osmolality, and sodium were measured. Aliquots were stored at −80°C for analysis of PGE2 and AQP2.

Blood pressure and heart rate (HR) was monitored by cuff every hour with an automatic ambulatory blood pressure monitor (ABP Monitor 90207 ™ SpaceLabs Medical, Inc, Redmond, WA).

### Blood and urine analysis

Plasma and urine concentration of sodium, creatinine, estradiol, and testosterone were measured using routine procedures at the department of Clinical Biochemistry. Plasma and urine osmolality were measured using the freezing point depression method (Advanced^TM^ Osmometer model 3900, Advanced Instruments, Norwood, MA).

AVP was determined in plasma by radioimmunoassay (RIA) (Bie and Sandgaard 2000) using a highly specific AVP antibody (AB3096) as previously described (Emmeluth et al. 1994). AVP was extracted from plasma using Sep‐Pak ^®^ Plus C18 extraction cartridges (Waters Corporation, Milford, MA). The detection limit was 0.10 pg/mL plasma and the intra and interassay coefficients of variation were 7.7% and 10.6%, respectively.

Oxytocin was measured in plasma following extraction on Sep‐Pak ^®^ Plus C_18_ extraction cartridge by RIA using an oxytocin‐specific rabbit‐antibody kindly provided by Dr. M. Morris, Dayton, OH. The detection level was 0.9 pg/mL; inter and intraassay coefficients of variation were 11.0% and 7.5%, respectively.

A commercial enzyme immunoassay kit (Amersham Pharmacia Biotech, Little Chalfont, UK) was used to determine the PGE2 concentration in the urine without prior extraction. Interassay and intraassay were approximately 13% and 9%. The detection limit was 40 pg/mL.

AQP2 was measured directly in urine using radioimmunoassay (RIA). A modification of a previous described method was used (Saito et al. 1997; Pedersen et al. 2001). Detection limit was 16 pg/mL urine. The PGE2 and AQP2 measurements were performed at the Water and Salt Research Center, Aarhus University Hospital, Denmark.

### Calculations

On the basis of urine and plasma measurements, excretion rates (E) and clearances (C) were calculated for sodium, creatinine, osmoles, PGE2, and APQ2 using standard formulas. Solute‐free water reabsorption (T_C_ H_2_O) is the difference between osmolar clearance (Cosmol) and urine flow (Uflow). The clearance of creatinine was used as an estimate of glomerular filtration rate.

### Statistical analyses

To compensate for the variability in body weight in the study population urinary excretion rates are expressed per kg body weight. The results are presented as mean ± SEM. To compare the variation over time for the two groups, we used a mixed effect ANOVA with a subject specific random effect using time and group as fixed effects, as well as their interaction, if this was statistically significant (Wald's test). When quantifying the day‐night differences, we created an indicator variable (day at time points 8:00, 14:00, 20:00, and 23:00 and night at time points 2:00, 5:00, and 8:00), and used this variable in place of time in the mixed effect model. When appropriate, the data were log‐transformed to fit the statistical model. These data are presented as geometric mean with normal 95% confidence intervals. Home recordings were compared to the in‐patient study using Student`s *t* tests. Correlations were calculated using Pearson′s correlation. Nondetectable values were set to equal half the detection level. Results were considered significant at *P* < 0.05.

## Results

Table 1 shows the demographic data of the participants. There was no difference in age or BMI between the two groups.

**Table 1:** Demographic data of the participants

	Participants	BMI (kg/m^2^)	Age (years)
Women with OC	11	24.3 ± 0.7 Range (21.4–27.0)	23.6 ± 2.3 Range (21–28)
Natural cycling women (NC)	15	22.7 ± 0.6 Range (18.7–27.9)	25.5 ± 2.8 Range (23–33)

Values are mean ± SEM.

### Hormones and plasma values

**Figure 1:**
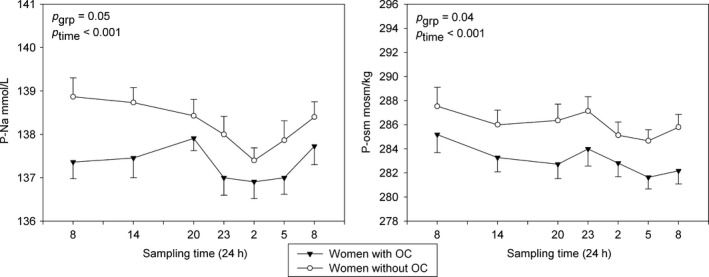
P‐sodium and p‐osmolality in women who uses OC (black triangles) and in the natural cycling women in midfollicular phase (white circles). Values are mean ± SEM.

Plasma sodium, osmolality, and creatinine all displayed a significant diurnal variation (*P* < 0.001). OC users showed significantly lower plasma osmolality (Δosm 3.05 ± 0.29 mosm/kg, *P* = 0.04) and plasma sodium (ΔNa^+^: 0.92 ± 0.09 mmol/L, *P* = 0.05) than the natural cycling women (Fig. 1). Packed cell volume was similar between the groups (*P* = 0.54). Clearance of creatinine was calculated as an estimate of GFR and corrected for body weight; the values were similar (*P* = 0.38) and showed no day to night variation (*P* = 0.19).

**Figure 2:**
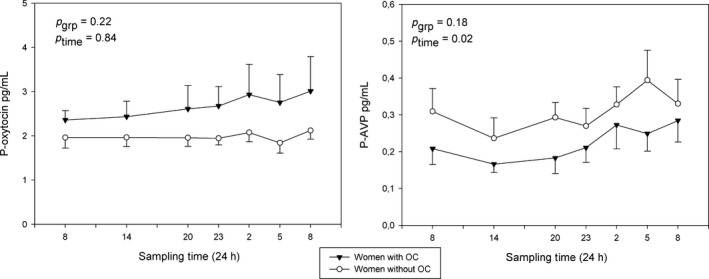
Diurnal rhythm of oxytocin and AVP in natural (white circles) and suppressed cycles (black triangles). Values are mean ± SEM.

There was no significant difference in *P*‐AVP between the groups (*P* = 0.18), but both groups had a significant nighttime increase (*P* = 0.02). For AVP, nocturnal increases of approximately 34% (95%‐CI: 15–56%) were found for both groups (*P* < 0.001). *P*‐oxytocin did not differ between the groups (*P* = 0.22) or over time (*P* = 0.83) (Fig. 2). The concentration of endogenous progesterone (day 1, 0800 h) was higher in the natural cycling women than in OC users (3.15 ± 0.25 nmol/L vs. 1.99 ± 0.15 nmol/L, *P* < 0.001). There was no difference in testosterone between the groups (1.55 ± 0.11 vs. 1.24 ± 0.12 nmol/L, *P* = 0.07). Endogenous estrogen was higher in the natural cycling women (0.30 ± 0.04 vs. 0.09 ± 0.01 nmol/L, *P* < 0.001).

### Diuresis

**Figure 3:**
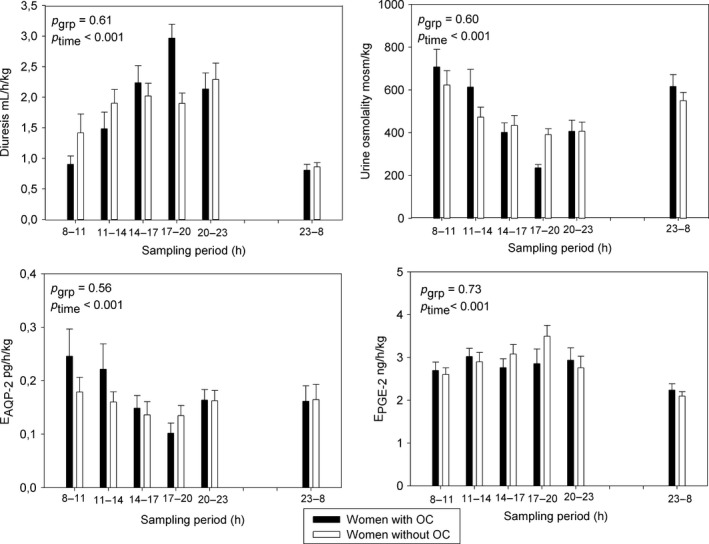
Diurnal rhythm of urine osmolality, diuresis, and excretion rates of PGE‐2 and AQP‐2 in women without intake of OC (white circles) or in OC users (black triangles). Bars are mean ± SEM.

Diuresis and urine osmolality showed diurnal rhythms. Diuresis peaked in late afternoon (OC users) or evening (NC) and showed minima at night. The observed nighttime reductions in urine production were highly significant (*P* < 0.001) and remarkably similar in the two groups (NC women: daytime: 1.90 ± 0.15 mL/(kg·h), night: 0.86 ± 0.07 mL/(kg·h) and OC users daytime: 1.94 ± 0.12 mL/(kg·h), night: 0.80 ± 0.10 mL/(kg·h). A diurnal rhythm in urine osmolality was found in both groups (*P* < 0.001), but there was no difference between groups (*P* = 0.60). Focusing on the day‐to‐night variation; urine osmolality increased significantly in the OC group (daytime: 473 ± 32 mosm/kg, night: 616 ± 55 mosm/kg, *P* = 0.03). In the NC group, the nighttime increase did not quite reach statistical significance (daytime: 465 ± 24 mosm/kg, night: 550 ± 38 mosm/kg, *P* = 0.07). Excretion of sodium displayed diurnal variation (*P* < 0.001) as well, and were similar in both groups (*P* = 0.21). There was no difference in the excretion of AQP2 (*P* = 0.56) or PGE2 (*P* = 0.73) between the groups. Both had diurnal variation (*P* < 0.001), but only PGE2 displayed lower nighttime excretion (*P* < 0.001). (Fig. 3). We found a significant positive correlation between AQP‐2 and urine osmolality (*r* = 0.4525, *P* < 0.001) and reabsorption of free water (*r* = 0.44, *P* < 0.001), but no correlation between AQP‐2 excretion and secretion of AVP. There was a negative correlation between 24 h diuresis and AVP (*r* = −0.25, *P* < 0.005). A similar correlation with oxytocin was not seen.

### Hemodynamics

**Figure 4:**
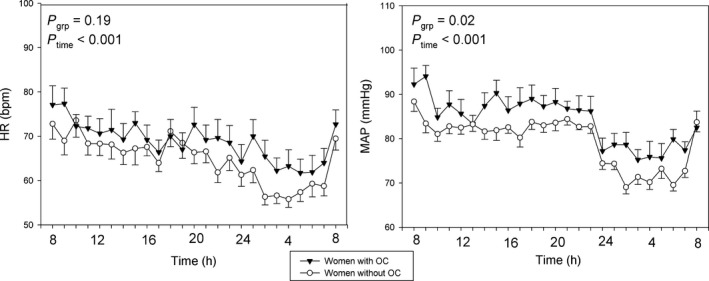
Mean arterial blood pressure (MAP) and heart rate (HR) in young women with natural (white circles) and suppressed cycles (black triangles). Values are mean ± SEM.

MAP was significantly higher in the women using oral contraceptives (OC: +4.7 ± 0.4 mmHg, *P* = 0.02). The nighttime dip in MAP was evident and similar in both groups (night: −10.3 ± 0.2 mmHg, *P* < 0.001) (Fig. 4). The diurnal variation in heart rate (HR) showed lowest values at night (Night: −7.9 ± 0.1 bpm, *P* < 0.001). We found no difference in HR between the groups (*P* = 0.19).

### Home recordings

The 24 h fluid‐intakes at home were similar in the two groups (30.9 ± 2.0 and 29.3 ± 2.3 mL/kg body weight in NC and OC women, respectively). The fluid intake at home did not differ from the fluid intake in the inpatient studies (NC women: *P* = 0.60, OC: *P* = 0.70). Twenty‐four‐hour urine volume during the in‐patient study was significantly higher than in the home recordings in both women and in OC users (*P* < 0.05). The day to night ratios were, however, similar (NC women: inpatient: 4.1 ± 0.5 vs. home: 3.9 ± 0.4, *P* = 0.7, OC: in‐patient: 4.8 ± 0.8 vs. home: 3.2 ± 0.4, *P* = 0.08).

## Discussion

In this study, we have been investigating whether the enhanced sex‐hormone concentrations obtained with OC use affect the diurnal regulation of urine production with focus on the relationship between AVP, oxytocin, AQP2, and PGE2.

Our study demonstrates that OC do not significantly change the overall concentration or the circadian profile of AVP, but confirms that high estrogen decreases both plasma sodium and plasma osmolality reflecting the resetting of the osmoreceptor for AVP release. There is no diurnal rhythm in oxytocin, which does not seem to be involved in renal regulation of fluid under normal physiological conditions. A significantly higher blood pressure was seen in the OC users. The urinary excretion of sodium, AQP2, and PGE2 were similar in the two groups.

We were not able to show a statistically significant difference in baseline AVP values, but did find a preserved nighttime increase in both groups. Baseline AVP levels have been reported to be unchanged (Stachenfeld et al. 1999; Calzone et al. 2001) or decreased (Bossmar et al. 1995) in women taking OCs. Previously, it seems that only one group has focused on diurnal investigations. They demonstrated an attenuated nighttime increase in AVP in women taking OCs (Kostoglou‐Athanassiou et al. 1998). The discrepancy between the studies may be due to different types and concentrations of estrogen and progesterone in the OCs used and probably the length of treatment as well. The participants in our study were all long‐term (>1 year) users.

The resetting of the osmoreceptor function to a lower osmotic threshold for AVP release is a well‐known phenomenon and occurs when estrogen is high (Stachenfeld et al. 1999; Calzone et al. 2001). This is confirmed in our study. We found no difference in urine production or urine osmolality between groups and can conclude that the production of urine is unaffected by this resetting, as indicated by other studies as well (Stachenfeld et al. 2003)

The influence of oxytocin on diuresis has been investigated in both humans and animals. Evidence supports an antidiuretic effect of oxytocin (Rasmussen et al. 2004; Joo et al. 2004). In rats it has been documented that oxytocin can function as an antidiuretic hormone at physiological plasma levels (Chou et al. 1995). Infusion of oxytocin in healthy young males led to a rise in plasma oxytocin to 50 pg/mL and resulted in an antidiuretic effect, which was mediated by the vasopressin receptor, albeit with a much lower affinity (Joo et al. 2004). Another previous study has demonstrated an increase in p‐oxytocin in women taking OC (Kostoglou‐Athanassiou et al. 1998) but whether this administration would increase plasma oxytocin to a level where it would exert antidiuresis remains undocumented. We did not observe diurnal variation in the concentration of oxytocin, nor did we show any difference between the groups. Plasma levels stayed low, between 2 and 4 pg/mL and there were no correlations to urine production. In summary, oxytocin does not play a role in diurnal urine formation under normal physiologic conditions, and intake of OC will not increase the plasma concentration to levels inducing an antidiuresis.

AVP and oxytocin exert their antidiuretic effect through a binding to the V_2_ receptor in the principal cells in the collecting ducts, thereby inserting AQP‐2 water channels, of which some subsequently will be excreted in the urine (Nielsen et al. 1993; Wen et al. 1999; Rai et al. 1997). Under normal physiological conditions we would anticipate elevated excretions of AQP‐2 under maximal antidiuresis during the night (Al‐Dameh et al. 2003). The lack of a nighttime increase in AQP‐2 excretion in this study may be due to the study design. We focused on the diurnal rhythm of hormones regulating the urine production. Healthy volunteers do not normally have nocturnal micturition and the increase in AQP‐2 excretion following the nocturnal rise in AVP would easily be blurred due to overnight sampling. Elucidation of the true circadian profile under normal physiologic conditions would require a urethral catheter and subsequent analysis of the hourly excretion of AQP‐2 during the night. However, the associated discomfort may disrupt the sleep and thus adversely affect the diurnal rhythm.

Urinary excretion of PGE‐2 revealed a distinct diurnal rhythm in excretion, but these were similar in the two groups. Gonadotropin‐induced ovulation with a subsequent increase in estradiol will lead to elevation of the urinary PGE‐2 excretion (Moutquin et al. 1984). This has led some authors to suggest that a p‐estradiol above normal range will result in increased PGE‐2 excretion (Stachenfeld et al. 2003). But what if estrogen is combined with progesterone as in oral contraceptives? In a study in postmenopausal women, estrogen and progesterone in combination, decreased the excretion of PGE2 (Akgul et al. 1998). Our participants used OC's and the content of both estrogen and progesterone can be the reason for the rejection of our hypothesis and it is from our study not possible to make any definite conclusions.

Mean arterial blood pressure was slightly, but significantly increased in the women taking oral contraceptives. Differences in body weight cannot explain the difference as the women had similar BMI. A mild rise in blood pressure is well‐known in connection with ingestion of oral contraceptives (Oelkers et al. 1995) even though it was more marked using the old formulations with higher content of estrogen (Boldo and White 2011). Causes of BP increase in women taking oral contraceptives are multiple and complex, but activation of the renin–angiotensin system (RAS) is involved (Boldo and White 2011). Ingestion of oral contraceptives with a high level of estrogen will result in an increased sympathetic tone and subsequently in a higher blood pressure (Minson et al. 2000; Carter et al. 2009). Progesterone is a mineralocorticoid antagonist and has marked natriuretic effect. The synthetic progesterone in the oral contraceptives are devoid of the antimineralocorticoid effect and cannot counteract the sodium retaining properties of estrogen (Oelkers 1996). As a consequence, OC may promote sodium and water retention and subsequently increase blood pressure.

We were not able to demonstrate a decrease in sodium excretion in the OC group compared to non‐OC users. However, the participants were in steady state with regard to sodium handling and as the experimental setup is observational, we would not expect to see any difference. Former studies, in which low‐dose oral contraceptives were used to elucidate the fluid balance, have shown a significantly decreased sodium excretion when estrogen was high (Stachenfeld et al. 1999; Calzone et al. 2001). One explanation of this finding may be that these studies are characterized by short‐term use of oral contraceptives (often only one cycle) and use the volunteers as their own controls.

To ensure that the in‐patient setup was comparable to the participant's everyday life a home study was conducted. Observational studies are influenced by the surroundings. Participants are admitted to the hospital for 24 hours, an intravenous cannula is inserted in their forearm and blood samples are taken throughout the day and night. This may change the sleep pattern and thereby the diurnal water handling. The fluid intake was similar at home and during the admission. The urine production was higher during the in‐patient investigation, but the day to night ratios were similar. Despite this reservation concerning the investigational circumstances, we found that the inpatient setup can be used to describe the everyday diurnal rhythm in urine formation.

The aim of this study was to describe the diurnal rhythm in female renal water handling under influence of oral contraceptives. Focus has been on AVP, oxytocin, AQP2, and PGE2. OC resets the osmoreceptors for AVP release and increases the blood pressure, whereas all other variables were similar between the groups, and it seems as if oral contraceptives do not alter the diurnal urine regulation.

However, this design has some limitations. All participants were preconditioned with fluid before admission, but we have not controlled for food (sodium) intake at home. They were asked to refrain from hard physical activity the last 2 days prior to admission, but we actually do not know if all have followed these rules, and the subjects could be either dehydrated or overhydrated as they arrived at the hospital. During the hospital stay, however, they were assigned to strict rules concerning fluid and foodintake.

Furthermore, the hormones in the follicular phase are not at all stable and the progestins in the OC have some androgenic properties compared to the endogenous progesterone. As the investigation has been done some years ago, all participants were at that time treated with oral contraceptives from the third generation containing 20 *μ*g ethinylestradiol and either gestodene (7) or desogestrel (4). The fact that we used two different progestins could have an impact on the results. However, both are highly potent with a low androgenic activity and serum levels and pharmacokinetics of the two substances are comparable (Stanczyk 1997). Due to the modest number of participants, it is not possible to subdivide the groups. This means that it is not possible to discuss the isolated effect of high estrogen, but only the overall effect of third generation of OCs on diurnal urine production. To elucidate an independent effect of estrogen are highly warranted. This will, however, require a completely different design.

## Conflicts of Interest

No conflicts of interest, financial or otherwise, are declared by any of the authors.
